# Supplementary material for: A novel ψ–χ fusion protein for unravelling the contributions of χ to DNA replication and repair
Source: Nucleic Acids Res. 2026 May 27;54(10):gkag533. doi: 10.1093/nar/gkag533 (PMC13213242; doi:10.1093/nar/gkag533)
Supplement: gkag533_Supplemental_Files [file gkag533_supplemental_files.zip › SupplementalFigures051226.pdf]

A Novel ψ-χ Fusion Protein for Unravelling the Contributions of χ to DNA Replication and Repair

AUTHORS

Kaylie A. Padgett-Pagliai[1], Jacob D. Grant[2], Matthew J. Petrides[1], Elijah S. P. Newcomb[1], Susan T. Lovett[2], Linda B. Bloom[1,*]

[1] Department of Biochemistry and Molecular Biology, University of Florida, Gainesville, FL, 32610-0245, USA
[2] Department of Biology, Brandeis University, Waltham, MA, 02453-9110, USA

* To whom correspondence should be addressed. Tel: +1 352 294 8379; Fax: +1 352 392 2953; Email: lbloom@ufl.edu

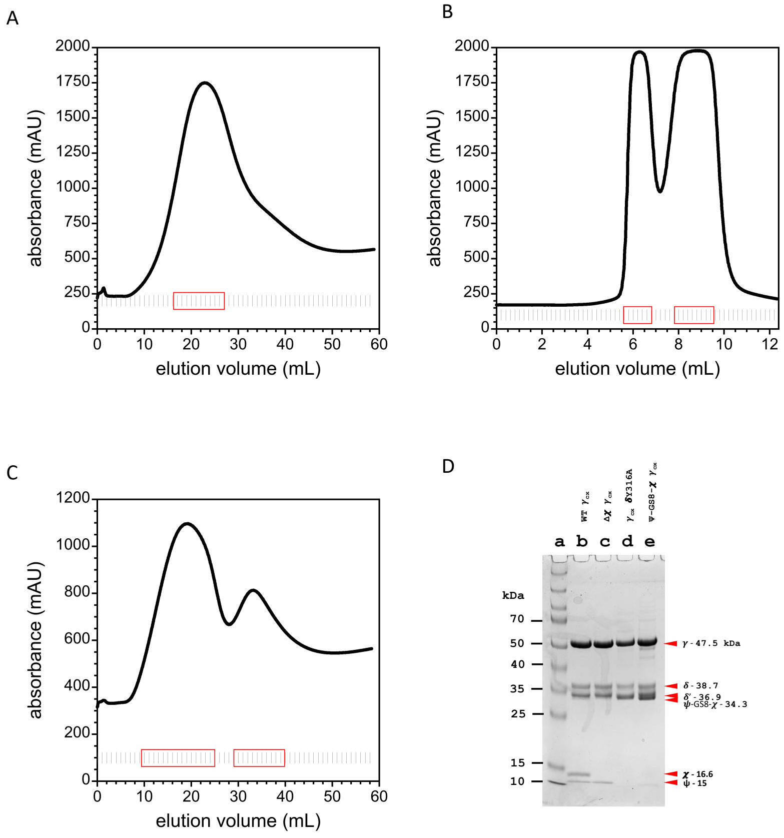

Supplemental Figure 1: Purification of fusion proteins. UV absorbance profiles for ψ-GS12-χ from HisTrap FF (A) and SP HP (B) columns are shown, with collected fractions indicated by vertical lines and pooled fractions by red boxes. The UV absorbance profile for ψ-GS8-χ from the HisTrap FF column is shown in (C), with fractions indicated as above. All chromatograms are representative. (D) SDS-PAGE analysis of purified proteins includes ψ-χ fusion assembled into the clamp loader, with wild-type clamp loader and clamp loader lacking χ as controls.

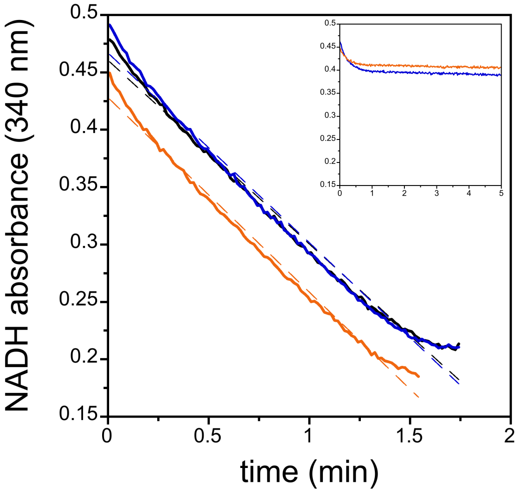

Supplemental Figure 2: DNA-dependent ATPase activity of ψ-χ fusions. The NADH absorbance at 340 nm as a function of time for an ATPase-coupled assay of γ complex variations on a 30-nt dsDNA with a 30-nt ssDNA overhang. 80 nM of each γ complex, WT γ complex (black), ψ-GS12-χ (blue), and ψ-GS8-χ (orange), were used to measure the decrease in NADH absorbance. Slope was fit to a line (R^2 = 0.98, 0.98, and 0.99, respectively, in dashed lines) to obtain a rate from the slope (-0.159 min^{-1}, -0.165 min^{-1}, and 0.169 min^{-1}, respectively) with rates of ATP hydrolysis at 25 μM min^{-1}, 27 μM min^{-1}, and 27 μM min^{-1}, respectively. Control reactions, each fusion γ complex with no DNA, are superimposed in the upper right. Rates of ATP Hydrolysis were calculated using Equation 2 in Materials and Methods.

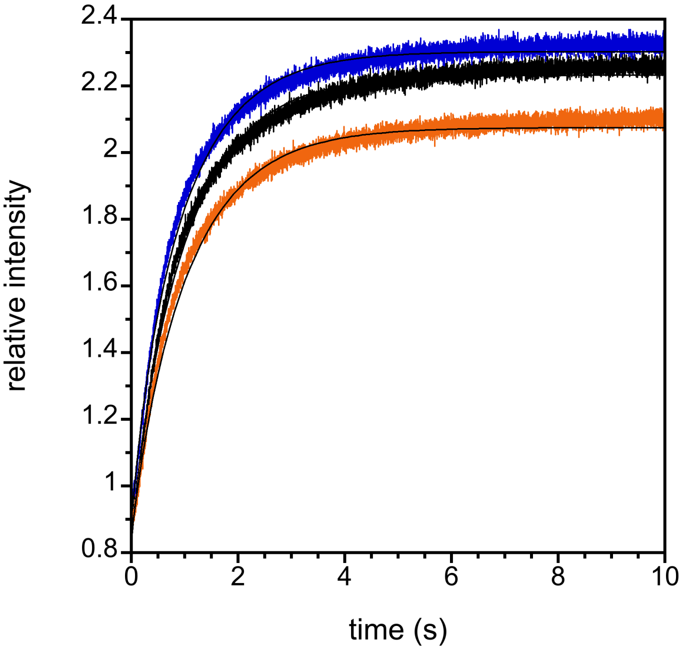

Supplemental Figure 3: Fusion clamp opening. Clamp opening reactions containing 60 nM γ complex clamp loader, 40 nM β-TMR_2, and 0.5 mM ATPγS. Gamma complex variations are shown as WT (black), ψ-GS12-χ (blue), and ψ-GS8-χ (orange). Solid black lines through the data show fits to a double exponential (Equation 3). n = 1

Supplemental Table 1. Strains and plasmids used in this study

**Strains**	**Relevant genotype**	**References**
Escherichia coli DH5α	F^- φ80*lac*ZΔM15 Δ(*lac*ZYA-*arg*F)U169 *rec*A1 *end*A1 *hsd*R17(r_K^-, m_K^+) *pho*A *sup*E44 λ^- *thi*-1 *gyr*A96 *rel*A1	Laboratory stock
E. coli BL21 (DE3)	F^- *omp*T *gal dcm lon hsd*SB(r_B- m_B-) λ(DE3 [*lac*I *lac*UV5-T7 *gene* 1 *ind*1 *sam*7 *nin*5])	Laboratory stock
E. coli BW25113	*Δ(araD-araB)567*, *ΔlacZ4787*(::rrnB-3), λ^-, *rph-1*, *Δ(rhaD-rhaB)568*, *hsdR514*	KEIO collection
E. coli BW25113 Δ*holC* (JW4216)	*Δ(araD-araB)567*, *ΔlacZ4787*(::rrnB-3), λ^-, *rph-1*, *Δ(rhaD-rhaB)568*, *ΔholC732::kan*, *hsdR514*	KEIO collection
Saccharomyces cerevisiae AH109	*MATa, trp1-901, leu2-3, 112, ura3-52, his3-200, gal4Δ, gal80Δ, LYS2 : : $GAL1_{UAS}$-$GAL1_{TATA}$-HIS3, $GAL2_{UAS}$-$GAL2_{TATA}$-ADE2, URA3 : : $MEL1_{UAS}$-$MEL1_{TATA}$-lacZ*	Matchmaker™ GAL4 Two-Hybrid System 3, TakaraBio
**Plasmids**	**Characteristics**	**References**
pET15-b_*holD*-GS12-*holC*	Expression vector, T7 promoter with *lac* operator, Amp^R, ψ-GS12-χ fusion synthesized by GenScript	GenScript Addgene 254005
pET15-b_*holD*-GS8-*holC*	Expression vector, T7 promoter with *lac* operator, Amp^R, ψ-GS8-χ fusion synthesized by GenScript	GenScript Addgene 254006
pETDuet-1_*holB*	Expression vector, T7 promoter with *lac* operator, Amp^R, *holB* cloned into MCS2	22 Addgene 254007
pETDuet-1_*holD*-GS8-*holC_holB*	Expression vector, T7 promoter with *lac* operator, Amp^R, *holD*-GS8-*holC* cloned into MCS1 and *holB* cloned into MCS2	This manuscript Addgene 254008
pETDuet-1_*holD*-GS12-*holC_holB*	Expression vector, T7 promoter with *lac* operator, Amp^R, *holD*-GS12-*holC* cloned into MCS1 and *holB* cloned into MCS2	This manuscript Addgene 254009
pCOLADuet-1_*holA_dnaX*	Expression vector, T7 promoter with *lac* operator, Kan^R, *holD*-GS8-*holC* cloned into MCS1 and *holB* cloned into MCS2	22 Addgene 254010
pUC57_*holD*-GS12-*holC*	Cloning vector, Amp^R	GenScript Addgene 254011
pBAD18	Expression vector, arabinose promoter, Amp^R	NovoPro
pBAD18_*holD*	Expression vector, arabinose promoter, Amp^R, SD sequence and gene cloned between KpnI and SphI	GenScript Addgene 254013
pBAD18_*holC*	Expression vector, arabinose promoter, Amp^R, SD sequence and gene cloned between KpnI and SphI	GenScript Addgene 254014
pBAD18_*holD_holC*	Expression vector, arabinose promoter, Amp^R, SD sequence upstream of each gene, separately, and cloned between KpnI and SphI	GenScript Addgene 254015

pBAD18_*holD*-GS12-*holC*	Expression vector, arabinose promoter, Amp^R, SD sequence and fusion cloned between KpnI and SphI	GenScript Addgene 254016
pBAD18_*holD*-GS12-*holC*_R128A	Expression vector, arabinose promoter, Amp^R, SD sequence and fusion cloned between KpnI and SphI. Mutation R128A in *holC* gene.	This manuscript Addgene 254017
pCL1	One plasmid strong positive yeast 2 hybrid control containing the full-length Gal4 transcriptional activator	Matchmaker™ GAL4 Two-Hybrid System 3, TakaraBio
pGADT7-T, pGBKT7-53	Two plasmid positive yeast 2 hybrid control that demonstrates a positive interaction of SV40 large T-antigen and murine p53	Matchmaker™ GAL4 Two-Hybrid System 3, TakaraBio
pGADT7-T, pGBKT7-lam	Two plasmid negative yeast 2 hybrid control that demonstrates a negative interaction of SV40 large T-antigen and Lamin C	Matchmaker™ GAL4 Two-Hybrid System 3, TakaraBio
pGADT7GW-*yoaA*+	Expression of YoaA fused to the activation domain of the Gal4 transcriptional activator	ySTL362, S. Lovett
pGBKT7GW-*yoaA*+	Expression of YoaA fused to the binding domain of the Gal4 transcriptional activator	ySTL363, S. Lovett
pGADT7GW-*holC*+	Expression of χ fused to the activation domain of the Gal4 transcriptional activator	ySTL364, S. Lovett
pGBKT7GW-*holC*+	Expression of χ fused to the binding domain of the Gal4 transcriptional activator	ySTL366, S. Lovett
pGADT7GW-*yoaA*+, pGBKT7GW-*holC*+	Expression of YoaA fused to the activation domain and χ fused to the binding domain of the GAL4 transcriptional activator	ySTL368, S. Lovett
pGADT7GW-*holC*+, pGBKT7GW-*yoaA*+	Expression of χ fused to the activation domain and YoaA fused to the binding domain of the GAL4 transcriptional activator	ySTL369, S. Lovett
pGADT7GW-*yoaA* T620A, pGBKT7GW-*holC*+	Expression of YoaA T620A fused to the activation domain and χ fused to the binding domain of the GAL4 transcriptional activator	ySTL377, S. Lovett
pGADT7GW-*holC*+, pGBKT7GW-*yoaA* T620A	Expression of χ fused to the activation domain and YoaA T620A fused to the binding domain of the GAL4 transcriptional activator	ySTL378, S. Lovett
pGADT7GW-*yoaA* T620A	Expression of YoaA T620A fused to the activation domain of the GAL4 transcriptional activator	ySTL387, S. Lovett
pGBKT7GW-*yoaA* T620A	Expression of YoaA T620A fused to the binding domain of the GAL4 transcriptional activator	ySTL388, S. Lovett

pGADT7GW-*holC*+, pGBKT7GW-*ssb*+	Expression of χ fused to the activation domain and SSB fused to the binding domain of the GAL4 transcriptional activator	ySTL478, S. Lovett
pGBKT7GW-*ssb*+	Expression of SSB fused to the binding domain of the GAL4 transcriptional activator	ySTL1173, S. Lovett
pGADT7GW-*ssb*+	Expression of SSB fused to the activation domain of the GAL4 transcriptional activator	ySTL1197, S. Lovett
pGADT7GW-*ssb*+, pGBKT7GW-*holC*+	Expression of SSB fused to the activation domain and χ fused to the binding domain of the GAL4 transcriptional activator	ySTL1229, S. Lovett
pGBKT7GW-*holD*-GS12-*holC*	Expression of ψ-GS12-χ fused to the binding domain of the GAL4 transcriptional activator	ySTL1301, S. Lovett
pGADT7GW-*ssb*+, pGBKT7GW-*holD*-GS12-*holC*	Expression of SSB fused to the activation domain and ψ-GS12-χ fused to the binding domain of the GAL4 transcriptional activator	ySTL1351, S. Lovett
pGADT7GW-*holD*-GS12-*holC*	Expression of ψ-GS12-χ fused to the activation domain of the GAL4 transcriptional activator	ySTL1353, S. Lovett
pGBKT7GW-*holD*-GS12-*holC*, pGADT7GW-*yoaA*+	Expression of ψ-GS12-χ fused to the binding domain and YoaA fused to the activation domain of the GAL4 transcriptional activator	ySTL1355, S. Lovett
pGBKT7GW-*holD*-GS12-*holC*, pGADT7GW-*yoaA* T620A	Expression of ψ-GS12-χ fused to the binding domain and YoaA T620A fused to the activation domain of the GAL4 transcriptional activator	ySTL1357, S. Lovett
pGBKT7GW-yoaA+, pGADT7GW-*holD*-GS12-*holC*	Expression of YoaA fused to the binding domain and ψ-GS12-χ fused to the activation domain of the GAL4 transcriptional activator	ySTL1359, S. Lovett
pGBKT7GW-yoaA T620A, pGADT7GW-*holD*-GS12-*holC*	Expression of YoaA T620A fused to the binding domain and ψ-GS12-χ fused to the activation domain of the GAL4 transcriptional activator	ySTL1361, S. Lovett

Supplemental Table 2. Oligonucleotides by use		
	**Primer Name**	**Sequence (5′→3′)***
**Cloning Primers**		
pETDuet-1_holD-GS8-holC_holB	holD-GS8-holC_Fw	aactttaagaaggagatataGCAATGACATCCCGACGAG
	holD-GS8-holC_Rv	gacttaagcaTTATTTCCAGGTTGCCGTATTC
	T7_LacO_RBS_holB_Fw	ctggaaataaTGCTTAAGTCGAACAGAAAG
	T7_LacO_RBS_holB_Rv	tgctcagcggtggcagcagcTTAAAGATGAGGAACCGG
pETDuet-1_holD-GS12-holC_holB	holD-GS8-holC_Fw	aactttaagaaggagatataGCAATGACATCCCGACGAG
	holD-GS8-holC_Rv	gacttaagcaTTATTTCCAGGTTGCCGTATTC
	T7_LacO_RBS_holB_Fw	ctggaaataaTGCTTAAGTCGAACAGAAAG
	T7_LacO_RBS_holB_Rv	tgctcagcggtggcagcagcTTAAAGATGAGGAACCGG
pBAD18_*holD*-GS12-*holC*_R128A	HolC_R128A_Fw	CTCTGAAACAACTGGCGGCCGAACGCTATAAAGCCTACC
	HolC_R128A_Rv	GGTAGGCTTTATAGCGTTCGGCCGCCAGTTGTTTCAGAG
**Linkers**		
GS8 sequence	GGSGSGGG	GGAGGCTCAGGAAGTGGTGGGGCT
GS12 sequence	GGSGSGSGSGGG	GGAGGTAGCGGAAGTGGCTCAGGGTCTGGGGGTGCG
**ATPase Assay**		
	SWP1	GGCCAGAATGTCCCTTTTAT
	YA1comp20-T65	TTTTTTTTTTTTTTTTTTTTTTTTTTTTTTTTTTTTTTTTTTTTTTTTTTTTTTTTTTTTTTTTTTATAAAAGGGACATTCTGGCC
**Stopped-Flow**		
	YA1	GGCCAGAATGTCCCTTTTATTACTGGTCGT
	JH9T35	TTTTTTTTTTTTTTTTTTTTTTTTTTTTTTTTTTTTTACGACCAGTAATAAAAGGGACATTCTGGCC

*Lower letters were added by PCR amplification.
